# Knowledge, attitudes and practice of healthcare ethics and law among doctors and nurses in Barbados

**DOI:** 10.1186/1472-6939-7-7

**Published:** 2006-06-09

**Authors:** Seetharaman Hariharan, Ramesh Jonnalagadda, Errol Walrond, Harley Moseley

**Affiliations:** 1 School of Clinical Medicine & Research, The University of the West Indies, Queen Elizabeth Hospital, Barbados, West Indies

## Abstract

**Background:**

The aim of the study is to assess the knowledge, attitudes and practices among healthcare professionals in Barbados in relation to healthcare ethics and law in an attempt to assist in guiding their professional conduct and aid in curriculum development.

**Methods:**

A self-administered structured questionnaire about knowledge of healthcare ethics, law and the role of an Ethics Committee in the healthcare system was devised, tested and distributed to all levels of staff at the Queen Elizabeth Hospital in Barbados (a tertiary care teaching hospital) during April and May 2003.

**Results:**

The paper analyses 159 responses from doctors and nurses comprising junior doctors, consultants, staff nurses and sisters-in-charge. The frequency with which the respondents encountered ethical or legal problems varied widely from 'daily' to 'yearly'. 52% of senior medical staff and 20% of senior nursing staff knew little of the law pertinent to their work. 11% of the doctors did not know the contents of the Hippocratic Oath whilst a quarter of nurses did not know the Nurses Code. Nuremberg Code and Helsinki Code were known only to a few individuals. 29% of doctors and 37% of nurses had no knowledge of an existing hospital ethics committee. Physicians had a stronger opinion than nurses regarding practice of ethics such as adherence to patients' wishes, confidentiality, paternalism, consent for procedures and treating violent/non-compliant patients (p = 0.01)

**Conclusion:**

The study highlights the need to identify professionals in the workforce who appear to be indifferent to ethical and legal issues, to devise means to sensitize them to these issues and appropriately training them.

## Background

There has been growing public concern regarding the ethical conduct of healthcare professionals. This is often reflected as complaints about poor ethical conduct and an increasing use of litigation against healthcare practitioners.

Although ethics as applied to medical practice dates back to the ancient civilization by the symbolic adherence to the Hippocratic Oath, codes of conduct and laws regulating the profession are devised and updated from time to time [1]. These codes have been included in the health professionals' training curriculum in many countries, and there has been a growth in the number of ethicists and ethical committees. Despite this, complaints against healthcare professionals appear to proliferate. This may be a reflection of both an increased public awareness as well as the inappropriate practices by the healthcare professionals.

Traditional medical training offers little help in resolving the ethical dilemmas encountered by healthcare professionals. There are opinions that very few physicians trained in the Caribbean have been exposed to training in this important area of medical practice. However, on qualifying, healthcare professionals are expected to know about ethical practice during application of their skills [2].

There have been many reports stressing the importance of incorporating ethical and legal issues into medical curricula [3-5]. There is also an argument that doctors and nurses should be taught medical ethics simultaneously [6]. There are reports of unethical behavioural patterns of medical students and medical practitioners with patients as well as colleagues [7-9]. The value of both positive and negative role models in teaching medical ethics has been well recognized [10,11].

There are many recommendations to strategize the teaching of medical ethics, most of it emphasizing the importance of tailoring it to the needs of the particular society in which it would be relevant. Medical ethics teaching should also be all inclusive, such as the teaching the value of 'heart' over 'mind', the value of incorporating deontological concepts etc [12].

On the other side of the spectrum, teaching medical ethics as if it is a scientific body of knowledge could also be dangerous. This is because it may miss the individualistic perception of morality and ethics innate to every professional, which would have been constructed by one's own unique cultural, socioeconomic and geographical background [13]. Hence the curriculum of medical ethics should be tailored to the social and cultural background where it is taught.

In order to formulate ethical curriculum germane to every region, the first step may be to determine the current basic knowledge and attitudes of the healthcare practitioners in the region. There have been few standard yardsticks designed to measure what is known and practised, so that educational efforts may be better targeted [14]. Physicians and nurses work closely together for patient-care, but the professional relations between the two categories may have differences with respect to their attitudes towards patient-care [15]. With this background the present study is an attempt to elucidate the knowledge, attitude and practice of the physicians and nurses in relation to healthcare ethics and law in Barbados.

## Methods

A thirty item self-administered structured questionnaire about knowledge of law and ethics and the role of an ethics committee in the healthcare system was devised *de novo *and tested. It was made available to all levels of staff at the Queen Elizabeth Hospital in Barbados (a tertiary care teaching hospital) during April, May 2003. The questionnaire included a full range of response options, designed to identify the practitioner's knowledge, beliefs and attitudes towards patient care I relation to healthcare ethics and law. Prior to distribution of the questionnaire a pilot study was done with a select group of healthcare workers who were asked to fill out the questionnaire and return with comments and criticism. Minor changes were made to the final instrument. The questionnaire is given in the 'Appendix' section.

The initial part of the questionnaire consisted of demographics such as occupation, age, gender, the duration of work experience and the frequency of ethical or legal problems encountered in practice. The second part of the questionnaire comprised of questions regarding the importance of knowledge of ethics and law to work, the source of knowledge of ethics and law and the preference for consultation regarding an ethical or legal problem should it arise.

Questions were asked whether the respondent knew of the presence of an ethics committee in the institution. The respondent was asked if he/she knows about the role of the ethics committee and if the ethics committee satisfied its role. There were eight roles described for the ethics committee in the questionnaire and the respondents were given a choice of 'yes', 'no or 'not sure' to respond to this question (Appendix).

In the final part of the questionnaire, respondents were asked to answer questions on everyday ethical issues, if the respondent agrees or disagrees to statements concerning ethical conduct, autonomy, paternalism, confidentiality, informing patients about wrongdoing and relatives of patient condition, informed consent, treating non-compliant or violent patient, religious beliefs influencing the treatment, abortion and euthanasia. The respondents were required to answer if they agree or disagree to the statements made on these issues and the gradation of the response was provided in a Likert scale ranging from 1 to 5 (1-strongly disagree, 2-disagree, 3-not sure, 4-agree and 5-strongly agree) (Appendix).

Among the four hundred distributed questionnaires, 373 were returned, out of which nine questionnaires were incompletely filled and were not included for analysis. These respondents included all levels of staff in the Queen Elizabeth Hospital. The present paper analyses and compares exclusively the responses of physicians and nurses (n = 159) among the survey. Data were analysed using Statistical Package for Social Sciences (SPSS) – version 8 software. Descriptive analyses were done for all data; the attitudes towards practical ethical problems were compared between nurses and physicians using a Chi square test. A Phi and Cramer's V value was obtained to determine the strength of the difference in their opinions. Statistical significance was fixed at the level of p < 0.05.

## Results

A total of 159 respondents belonged to the category of either physicians or nurses. Interns, post-graduate medical residents, senior house officers and registrars were considered as junior physicians and the rest falling into the category of consultant physicians. 47% of the respondents were physicians, and 53% were nurses including sisters-in-charge.

Table 1 shows the demographics of physicians and nurses who responded to the questionnaire. There were more female nurses and more male consultant physicians consistent with the general trend.

**Table 1:** Demographics of respondents

**Category**	**Number (%)**	**Gender ratio (M:F)**
Junior physicians	48 (30%)	1.2: 1
Consultant physicians	27 (17%)	4.4: 1
Nurses	64 (40%)	1: 15
Sisters-in-charge	20 (13%)	0: 1

The age distribution of the respondents was also consistent with the categories of medical and nursing staff. Work experience stretched across the entire spectrum of 1 year to over 31 years and was consistent with the occupations and ages of respondents. 90% of junior physicians were in the age group of 20–29 years and 65% of them had 4–10 years work experience. 72% of staff nurses were in the age group of 30–49 years and 60% of them had work experience of 7–20 years. 70% of consultant physicians and 80% of sisters-in-charge had a work experience of 20 years and more.

**Figure 1:**
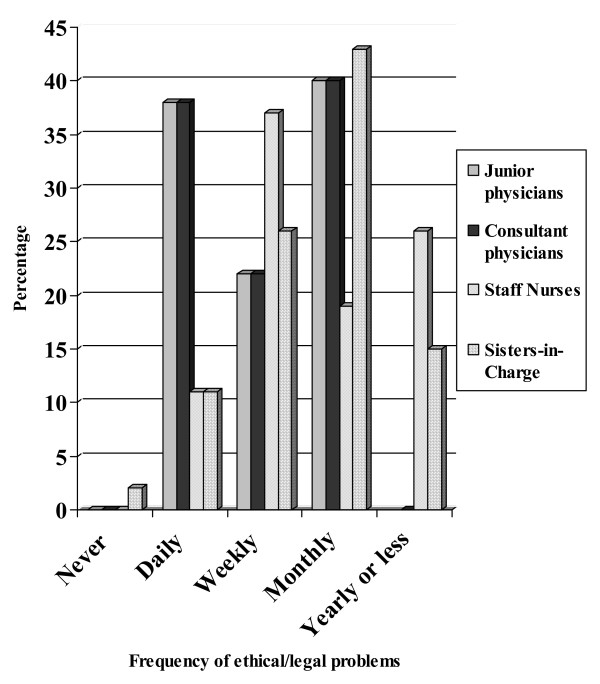
Frequency of ethical or legal problems encountered.

Figure 1 shows the responses to the frequency of ethical and legal problems encountered by the physicians and nurses. There were more physicians than nurses who encountered these problems on a daily and monthly basis and more nurses than physicians who encountered them on yearly basis. Some of the sisters-in-charge responded that they never encountered such a problem.

All the physicians and 90% of nursing staff responded that knowledge of ethics is important to their work. Only one nurse responded that knowledge of ethics was "not at all important". There was a good correlation in that those who responded that they saw ethical problems every day never responded that ethical knowledge was unimportant.

**Figure 2:**
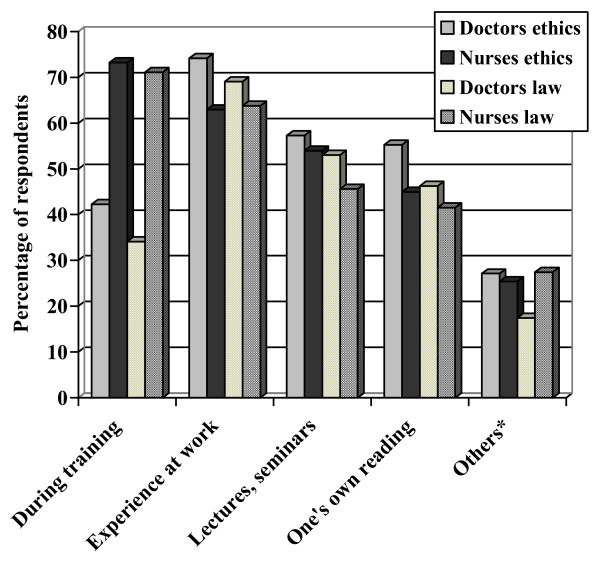
**Source of knowledge of healthcare ethics and law**. * Church, court reports etc.

Figure 2 shows the sources of knowledge regarding medical ethics and law. More than half of the respondents answered that they acquired their knowledge of ethics and law from multiple sources. More number of nurses than physicians responded that they acquired their knowledge of ethics and law during training. More than 70% of physicians and nurses responded that they acquired their knowledge of ethics during work.

A little more than half of both physicians and nurses responded that they had "no" or 'little' knowledge of the law; 45% of sisters-in-charge said they knew most of the law pertaining to work.

Among the 11% of physicians who did not know the main contents of the Hippocratic Oath, there were 5 junior physicians, one consultant physician and one General Practitioner. Sixty percent of sisters-in-charge also responded that they knew the contents of the Hippocratic Oath. However, 34% nurses and one sister-in-charge did not know the "Nurses Code". Over 90% physicians and nurses did not know of the Nuremberg Code or the Helsinki Declaration.

Tables 2 and 3 show the preferences of physicians and nurses as to whom to approach when faced with an ethical and legal problem. While majority of the nurses said they would approach the matron, majority of physicians said they would approach the immediate supervisor.

**Table 2:** Preference in consulting on an ethical problem

**Whom to consult**	**Physicians (%)**	**Nurses (%)**
Colleague	58	35
Supervisor	47	33
Head of Department	49	51
Chief of Medical staff	23	0
Matron	0	40
Hospital Administrator	8	29
Ethics Committee	36	17
Professional Association	20	17
Priest	8	33
Text, Internet	13	4
Close friend/family	9	25

**Table 3:** Preference in consulting on a legal problem

**Whom to consult**	**Physicians (%)**	**Nurses (%)**
Colleague	31	31
Supervisor	40	32
Chief of Medical staff	20	0
Matron	0	39
Hospital Administrator	21	23
Professional insurance company	29	0
Trade Union	24	26
Lawyer	45	54

29% of physicians and 37% of nurses were unaware of the existence of an ethics committee at the institution. Of those who answered that there was a committee, many physicians felt that the committee is not fulfilling its role. 17% of physicians and 41% of nurses felt that the committee is performing its role satisfactorily. Among the physicians who were not aware of an ethics committee in the hospital, a quarter responded that there is a definite role for an ethics committee. Among those who were aware of an ethics committee at the hospital nearly half of physicians and nurses felt that its role is to advise the administration on ethics rules in the institution, to advice the staff and patients on ethical problems arising out of work and to teach ethical conduct. One-third of physicians and nurses felt that the role of an ethics committee is to ensure that research is conducted properly.

Table 4 depicts the responses of physicians and nurses regarding the various aspects of practicing ethics. There was a statistically significant difference between the opinions of physicians and nurses with respect to adherence to patients' wishes, confidentiality, paternalistic attitude of doctors, consent for procedures and treating violent/non-compliant patients. The doctors were stronger in their opinions than the nurses in these issues. There were no differences in the strength of the opinions regarding other issues such as informing patient regarding wrongdoing, informing close relative of a patient, seeking consent for children, abortion and euthanasia, where doctors and nurses were equally opinionated in these issues.

**Table 4:** Practice of ethics

**Issues in practice of medical ethics**	**Occupation**	**Disagree**	**Agree**	**Chi square**	**Cramer's V**	**p-value**
Patient's wishes must always be adhered to	*Physicians*	43	23	4.0	0.15	p = 0.03
	*Nurses*	65	26			
Patient should be always informed of wrongdoing	*Physicians*	11	68	0.23	0.09	p = 0.17
	*Nurses*	7	79			
Confidentiality – not important	*Physicians*	82	1	11.2	0.26	p = 0.001
	*Nurses*	76	14			
Doctor should do best irrespective of patient's opinion	*Physicians*	67	10	8.0	0.22	p = 0.004
	*Nurses*	61	28			
Consent only for operations – not for tests and medications	*Physicians*	72	6	3.7	0.15	p = 0.04
	*Nurses*	74	16			
Close relatives should always be told about patient condition	*Physicians*	62	17	2.6	0.12	p = 0.07
	*Nurses*	60	29			
Children should never be treated without consent of parent	*Physicians*	13	70	0.78	0.07	p = 0.25
	*Nurses*	10	80			
Doctors & nurses should refuse to treat a violent patient	*Physicians*	71	7	5.9	0.19	p = 0.01
	*Nurses*	67	20			
If law allows abortion, doctors cannot refuse to do abortion	*Physicians*	83	3	3.2	0.13	p = 0.07
	*Nurses*	78	9			
If a patient wishes to die, he or she should be assisted in doing so	*Physicians*	81	2	1.9	0.11	p = 0.15
	*Nurses*	80	6			
If patients refuses treatment due to beliefs, they should be instructed to find another doctor	*Physicians*	66	11	1.4	0.09	p = 0.17
	*Nurses*	77	7			

Table 5 shows the responses about the usefulness of the instruments to learn ethics and law. Panel discussions and workshops seemed to be useful instruments in most respondents.

**Table 5:** Instruments for learning ethics and law

**Instruments found useful**	**Physicians (%)**		**Nurses (%)**	
	
	**Juniors**	**Consultants**	**Staff nurses**	**Sisters-in-charge**
Ethics journals	58	63	43	45
Books on ethics	58	52	33	40
General texts	60	78	25	25
Media *(Newspapers/TV)*	57	80	29	33
Workshops	74	56	33	25
Lectures *(UG/CME)*	58	59	47	50
Panel discussions	71	59	43	30
Case conferences	19	37	33	30

UG = Undergraduate lectures

CME = Continuing Medical Education lectures

## Discussion

The findings of the present study clearly show the difference in the knowledge and attitudes between physicians and nurses regarding the medical ethics and law. The respondents were representative of different levels of physicians and nursing staff consisting of junior physicians inclusive of post-graduates, consultant physicians, nurses and sisters-in-charge and the responses were reflective of these categories.

The frequency of encountering the ethical and legal problems was a full spectrum ranging from "never" to "every day". Junior physicians and nurses responded that they encountered an ethical problem more often than the consultant physicians and sisters-in-charge, perhaps due to their more frequent contact with patients. The concern here is that it has to be assumed that although the junior staff had often encountered some form of ethical problem, it might not have been brought to the notice of the senior staff. Because the senior staff should act as mentors to their juniors, it is important that they should have been made aware of the problems that do arise. However it is unsure whether the juniors are perceiving problems where there are none. This may also imply that while offering training about law, ethics and the role of ethical committees, both the junior and senior staff needs to be included. If the senior staffs function autonomously during ethical dilemmas, even when they lack adequate knowledge of ethics, this may send wrong signals to the junior staff that adequate knowledge of ethics may be unnecessary for a successful practice [16].

Most of the respondents agreed to the importance of ethical knowledge, although one-tenth of the staff nurses did not think that it is important. Those respondents, who thought that the knowledge of ethics and law was unimportant, also responded that they never saw problems. Perhaps due to the poor awareness regarding ethics and ethical situations, many of these respondents would not have possibly recognized the problems at workplace.

Very few respondents had obtained their knowledge of ethics and law from a single source. It is also interesting to note that the source of knowledge of healthcare ethics and law amongst junior physicians during training appeared to be less important than the experience at work, lectures and seminars and one's own reading (2). This shows that the curricular training regarding ethics and law pertaining to work is either inadequate or ineffective. Traditionally, healthcare personnel receive limited training in formal ethics even though their daily work involves direct and often crucial intervention in others' lives [17]. The teaching of medical ethics was introduced as a distinct entity into the medical curriculum of the Faculty of Medical Sciences, The University of the West Indies in 1991, but this teaching has been didactic in a lecture theatre setting [18]. It has been stressed that teaching and training which commence at the start of the course of study in medical and other healthcare professional schools, should be an ongoing process akin to continuing medical and nursing education [19]. Since both physicians and nurses feel that their main source of knowledge of healthcare ethics and law was during experience at work, such job experiences should be used to reinforce ethical knowledge and practice.

Another major finding of the study was that the majority of the respondents did not know enough of the law pertaining to their workplace. Also, there were some physicians and nurses who did not know the contents of their respective codes. The fact that more than 90% of the respondents had no knowledge regarding the Nuremberg Code and or the Helsinki Declaration indicates that there is very little knowledge regarding the ethics of research.

Many of the respondents preferred to consult either their colleague, immediate supervisor or the head of their department for ethical and legal issues. Although many of them have registered with a trade union or a professional insurance company, less than one-third only opted to consult them when faced with a legal problem at work. This is consistent with the commonly preferred opinion to settle the matter in the departmental level rather than taking it farther into higher levels. It is interesting that two-thirds of physicians and one-third of nurses responded that they would consult a colleague despite the feeling that they knew little of the law. Does this reflect a separation of ethical from legal conduct in their minds or a discomfort with discussing problems with seniors? This study was not designed to answer that question. The relatively higher level of response from physicians and nurses that they would consult a lawyer on problems may reflect that the lawyers may be available as friends or relatives rather than any availability of funds for such consultation.

The unawareness regarding the ethical committee in the present study is very similar to another study regarding physicians' attitude and perceptions of a Hospital Ethics Committee from the United States, wherein a large number of professionals expressed dismay at the "invisibility" of the ethics committee [20]. Our Hospital Ethics Committee is currently not well known to many professionals and there is a need for making it aware to the staff of our hospital.

Responses from both medical and nursing practitioners to questions pertaining to practical ethics (Table 4) suggest that the majority of them were aware of the common ethical issues. The significantly stronger opinions of the doctors and the nurses in certain issues such as opinions of physicians and nurses with respect to adherence to patients' wishes, confidentiality, paternalistic attitude of doctors, consent for procedures and treating violent/non-compliant patients again may reflect the difference in the intensity of training between the two professionals.

On the question of autonomy there was wide difference of opinion among different cadres of medical and nursing staff. In another study on attitudes towards patient autonomy, UK nurses showed a greater commitment to patient autonomy than did any of the US groups, showing that there may be regional variations [21]. The fact that many senior level staff did not feel that the patient's wishes should be adhered to at all times, shows the lack of knowledge of the basic principles of medical ethics.

Ethical case conferences were helpful for many of the respondents to know about ethics. Case conferences are recent phenomena in our hospital and the proceedings of these case conferences are published and made available to all healthcare professionals.

## Conclusion

Physicians and nurses commonly encounter ethical and legal issues in their workplace. However, many of these professionals are either unaware of their importance or unable to appropriately deal with these issues. Since the findings of the study identify that learning at workplace has been valuable to gain knowledge about ethics and law, there is a need to identify those who appear to be indifferent to ethical and legal issues and devise means to sensitize them to these issues in the workplace. Practical education in ethics, particularly in a multidisciplinary setting, could assist in bridging the gap in ethical approaches between nurses and physicians.

## Competing interests

The author(s) declare that they have no competing interests.

## Authors' contributions

SH coordinated the study, interpreted the data, statistically analysed the data and drafted the manuscript. RJ conceived of the study, and participated in its design and coordination and revised the manuscript. EW participated in the design of the study and revised the manuscript. HM participated in the design of the study and revised the manuscript.

All authors read and approved the final manuscript.

## Pre-publication history

The pre-publication history for this paper can be accessed here:


